# Wor1 establishes opaque cell fate through inhibition of the general co-repressor Tup1 in *Candida albicans*

**DOI:** 10.1371/journal.pgen.1007176

**Published:** 2018-01-16

**Authors:** Selma S. Alkafeef, Clinton Yu, Lan Huang, Haoping Liu

**Affiliations:** 1 Department of Biological Chemistry, University of California, Irvine, California, United States of America; 2 Department of Physiology and Biophysics, University of California, Irvine, California, United States of America; Pacific Northwest Research Institute, UNITED STATES

## Abstract

The pathogenic fungus *Candida albicans* can undergo phenotypic switching between two heritable states: white and opaque. This phenotypic plasticity facilitates its colonization in distinct host niches. The master regulator *WOR1* is exclusively expressed in opaque phase cells. Positive feedback regulation by Wor1 on the *WOR1* promoter is essential for opaque formation, however the underlying mechanism of how Wor1 functions is not clear. Here, we use tandem affinity purification coupled with mass spectrometry to identify Wor1-interacting proteins. Tup1 and its associated complex proteins are found as the major factors associated with Wor1. Tup1 occupies the same regions of the *WOR1* promoter as Wor1 preferentially in opaque cells. Loss of Tup1 is sufficient to induce the opaque phase, even in the absence of Wor1. This is the first such report of a bypass of Wor1 in opaque formation. These genetic analyses suggest that Tup1 is a key repressor of the opaque state, and Wor1 functions via alleviating Tup1 repression at the *WOR1* promoter. Opaque cells convert to white *en masse* at 37°C. We show that this conversion occurs only in the presence of glycolytic carbon sources. The opaque state is stabilized when cells are cultured on non-glycolytic carbon sources, even in a *MTL***a**/α background. We further show that temperature and carbon source affect opaque stability by altering the levels of Wor1 and Tup1 at the *WOR1* promoter. We propose that Wor1 and Tup1 form the core regulatory circuit controlling the opaque transcriptional program. This model provides molecular insights on how *C*. *albicans* adapts to different host signals to undergo phenotypic switching for colonization in distinct host niches.

## Author summary

The opportunistic fungal pathogen *Candida albicans* is a common member of the human gut microbiota, but can also cause serious infections in immune-compromised individuals. Morphological plasticity is essential for its ability to adapt to and colonize a varied array of diverse host niches. One such morphological cell fate transition is between the pathogenic white state and the mating-competent, gut-colonizing, commensal opaque state. The opaque state is controlled by the master regulator Wor1. In this study we sought to identify how Wor1 functions to induce and maintain the opaque phase. Starting with a mass spectroscopy-based approach followed up with genetic analyses, we identified the general transcriptional repressor Tup1 as a key repressor of the opaque state and discovered Wor1’s function in repressing Tup1 activity. This regulation of opaque switching through inhibition of a repressor provides insights for how cell fate is regulated in response to different host cues and further explains how *C*. *albicans* can switch between commensal and pathogenic states.

## Introduction

*Candida albicans* is a common opportunistic fungal pathogen of humans. Found in the mouth, gastrointestinal tract, vagina and skin, *C*. *albicans* is harmless to the healthy, but in immunocompromised individuals *C*. *albicans* can cause serious infection, with a mortality rate of up to 40% in disseminated systemic infections [1]. *C*. *albicans* is able to transition between several phenotypic forms. This ability allows *C*. *albicans* to easily adapt to and inhabit various diverse host niches [1]. These different phenotypic states display not only different morphological features such as cell shape and size, but also altered metabolism and gene expression patterns. One such transition is the switch between the white and opaque states [2], which is regulated by many different stimuli such as N-acetylglucosamine [3, 4], high CO_2_ [5], hypoxia [6], temperature [7, 8], and genotoxic stress [9–11]. The opaque phase is the mating competent form of *C*. *albicans* and is inhibited by the **a**1-α2 repressor complex [12, 13]. In addition to genes related to mating, white and opaque cells show differential regulation of hundreds of genes involved in metabolism, adhesion, cell surface composition, stress response, signaling, and virulence [14, 15]. Opaque cells generally show reduced virulence compared to white cells in a systemic setting, however, opaque cells are excellent colonizers of the skin and show increased fitness in cutaneous infection models [16, 17]. Additionally, opaque cells are less efficiently phagocytosed than white cells, implicating phase switching in host immune evasion [18, 19]. More recently, white-opaque switching has been observed in *MTL***a**/α strains by passage through the mammalian gut [20] and in certain *MTL***a**/α clinical isolates [21]. These **a**/α opaque cells are somewhat distinct from their mating-competent *MTL* homozygous counterparts, and show enhanced commensalism compared to white cells in the gut [20].

On the molecular level, the opaque state is induced through expression of the master regulator Wor1, which regulates its own expression and forms a stable autoregulatory feedback loop to induce and maintain the opaque state [22–24]. Wor1 is part of an extensive underlying network of transcriptional regulators governing white-opaque switching [25–28]. While some regulators show differential phase expression, all identified white-opaque regulators show opaque-enriched binding at each other’s promoter regions [27]. This is most evident at the 8kb-long *WOR1* promoter, where both positive and negative regulators of the opaque phase share enhanced binding at the same regions along the active *WOR1* promoter. In addition, white-opaque switching is also regulated at the levels of promoter chromatin and protein translation [10, 11, 29–32]. Wor1 belongs to a class of conserved fungal transcription factors, many of which have been identified as master regulators of morphological development and virulence in pathogenic fungi, such as Ryp1 in the human pathogen *Histoplasma capsulatum* [33] and Sge1 in the plant pathogen *Fusarium oxysporum* [34]. This family of proteins contains an uncharacterized C-terminal domain and two unique, highly conserved N-terminal domains termed WOPRa and WOPRb, both of which are essential for the sequence-specific DNA binding properties of this class of protein [35–37]. Point mutation of these domains leads to disruption of Wor1 DNA binding and its ability to induce the opaque phase [36, 37]. How Wor1 functions to induce and sustain the opaque state is not known.

Transcriptional repression is frequently used to control gene expression in cell fate determination. The general repressor Tup1 is a member of the highly conserved Gro/TLE family of proteins that are present in many species across several kingdoms [38, 39]. It does not bind DNA directly but instead is recruited to different genomic regions through interactions with sequence-specific transcriptional regulators to repress many diverse sets of genes [40, 41]. Tup1 inhibits gene expression through several mechanisms, including recruitment of histone deacetylases, chromatin remodeling, and modulation of Mediator function [42, 43]. In this study we use a global mass spectroscopy-based approach to identify proteins associated with Wor1 in order to understand how Wor1 functions as the master regulator of the opaque phase. We identify the general co-repressor Tup1 as a Wor1-interacting protein and the key repressor of the opaque state. We further demonstrate that external cues, such as temperature and carbon source, regulate Wor1 and Tup1 at the *WOR1* promoter to control cell fate.

## Results

### Tandem affinity purification of proteins cross-linked to Wor1 identifies Tup1 complexes

**Fig 1:**
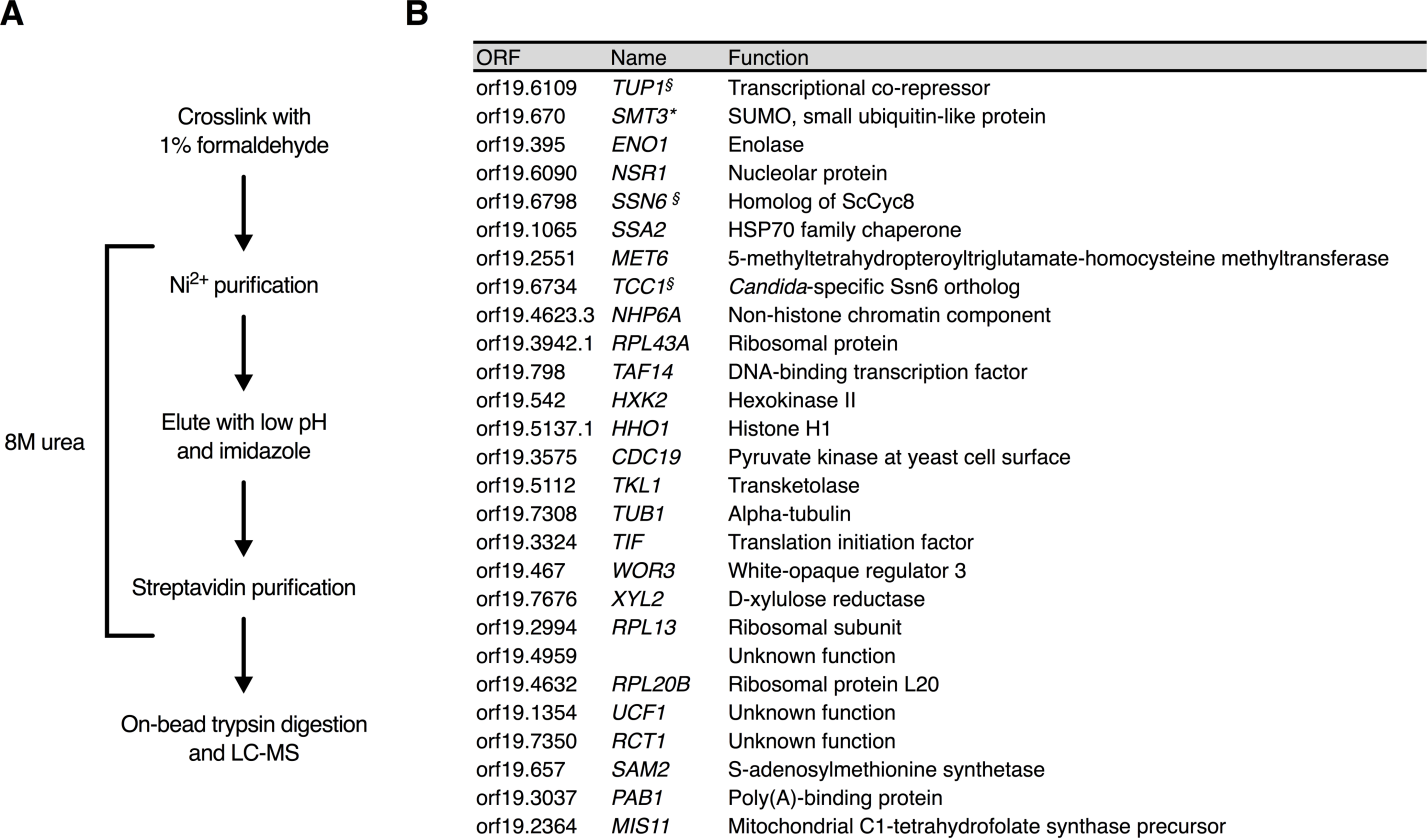
Identification of Wor1 interacting proteins by tandem affinity HBH purification coupled with mass spectroscopy. **(A)** The workflow for an HBH purification in denaturing conditions utilizing formaldehyde cross-linking. **(B)** A list of Wor1 interacting proteins compiled from five HBH purifications. All proteins listed were observed only in Wor1-HBH (HLY4532) purifications with crosslinking in three or more of the purifications performed. Proteins marked by § are known components of the Tup1 co-repressor complex. Proteins marked with an asterisk were also observed in Wor1-HBH purifications without cross-linking. A complete list of proteins identified in each Wor1-HBH purification is provided in S1 Table. Proteins are ranked in descending order first by number of times observed in independent purifications and then by average normalized iBAQ (intensity-based absolute quantification) value.

In an effort to identify key regulators and mechanisms governing white-opaque switching, we employed a biochemical approach to better understand how Wor1 functions as the master regulator of the opaque phase. We set out to identify Wor1 interacting proteins by utilizing cross-linked tandem affinity purification in denaturing conditions coupled with mass spectrometry using a His-Biotin-His (HBH) tag cloned onto the C-terminal end of Wor1 (Fig 1A). The HBH tag, originally designed for use in *S*. *cerevisiae*[44], was codon optimized for efficient expression in *C*. *albicans*, a member of the CTG clade [45]. The benefit of using this tandem affinity purification method is two-fold. Firstly, cross-linking allows us to capture weak and transient interactions that may otherwise be missed; secondly, the ability to perform both purification steps under denaturing conditions helps to prevent protein degradation and loss of post-translational modifications. *C*. *albicans* cells carrying a *MAL2* promoter driven copy of the C-terminally HBH-tagged Wor1 were grown in maltose-containing media overnight then cross-linked with 1% formaldehyde, washed, lysed in 8M urea buffer, and the resulting clarified protein extracts were incubated with Ni^2+^ agarose beads. The bound proteins were then eluted and incubated with streptavidin-conjugated agarose beads. Samples were treated to an on-bead trypsin digestion and the resulting peptides were analyzed by LC-MS (Fig 1A). As a negative control, the same procedure was performed without cross-linking to determine which proteins are bound without crosslinking, such as those covalently linked to Wor1 or non-specifically interact with beads. Another negative control was performed with cross-linking, but using a strain without HBH-tagged Wor1 to determine which proteins nonspecifically interact with the Ni^2+^- and streptavidin-conjugated agarose beads. Proteins found associated with Wor1-HBH in 3 out of 5 biological replicates were ranked by intensity-based absolute quantification (iBAQ) and are shown in Fig 1B [46, 47]. A complete list of identified proteins is provided in S1 Table.

The general transcriptional co-repressor Tup1 consistently ranked as the top Wor1-associated protein (Fig 1B). In *C*. *albicans*, the Tup1 co-repressor complex can exist in two forms, either with Ssn6 or with Tcc1, which are both homologs of *S*. *cerevisiae* Cyc8 [48–50]. Both Ssn6 and Tcc1 were top ranked in all 5 purifications (Fig 1B). Also observed were chromatin-associated proteins Non-Histone Protein 6A (Nhp6a), which is commonly associated with actively transcribed genes [51], and Taf14, which is a subunit of several complexes including the INO80, SWI/SNF, and NuA3 complexes (Fig 1B, S1 Table) [52]. The white-opaque regulator Wor3 was found in 3 out of 5 experiments. Although Wor3 is not known to interact with Wor1, both bind to same promoter regions of *WOR1* and other phase regulators [26]. Unlike other interacting proteins, Smt3 was identified as Wor1-associated in denaturing conditions without cross-linking (Fig 1B). This demonstrates that Wor1 is SUMOylated, which is consistent with a recent publication [53].

### Tup1 is bound to the *WOR1* promoter preferentially in opaque cells

**Fig 2:**
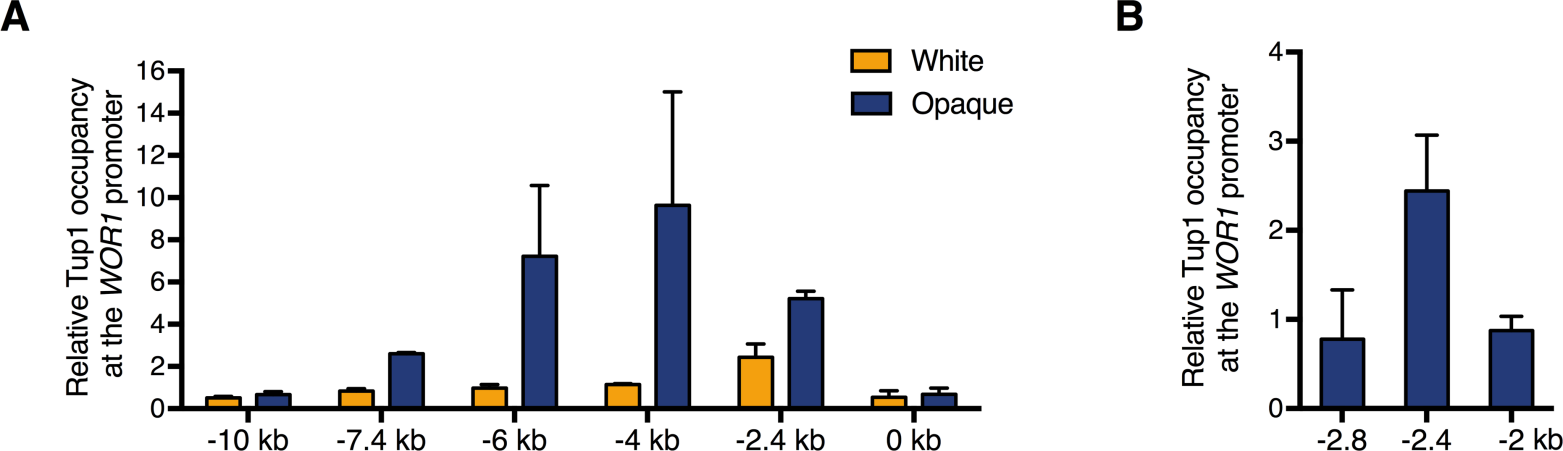
Tup1 binds along the *WOR1* promoter differentially in white and opaque phases. **(A)** ChIP of Tup1-HA in white and opaque cells. Overnight cultures of white and opaque cells of a wild-type strain (JYC5) and a strain carrying Tup1-HA (HLY4538) were diluted in SCD and grown to log phase at room temperature before formaldehyde. Enrichment is presented as a ratio of qPCR of the *WOR1* promoter IP (bound/input) over an *ADE2* control region IP (bound/input) of the tagged strain, further normalized to the control strain. Values are the average of three independent ChIP experiments with error bars representing the s.d. **(B)** Additional qPCR of Tup1 binding around -2.4kb upstream of the *WOR1* TSS in white cells from **(A)**.

To determine if Tup1 interacts with Wor1, we carried out immunoprecipitation experiments in native conditions without cross-linking in opaque cells. Despite many attempts, we were unable to observe a Wor1-Tup1 interaction in native conditions by IP with either Tup1-HA or Wor1-FLAG. Failure to observe binding in native conditions could mean that the interaction is too weak to capture by native IP or that the two proteins are brought to interact through association at the chromatin. To test the latter possibility, we performed ChIP with a C-terminal 3xHA tagged Tup1 in white and opaque cells, and examined Tup1 binding at several regions along the *WOR1* promoter where Wor1 and other key white-opaque transcriptional regulators have been shown to bind [27]. We found that Tup1 binds to the *WOR1* promoter at 7.4kb, 6kb, 4kb, and 2.4kb upstream of the *WOR1* TSS preferentially in opaque cells despite having the same levels of Tup1 protein in both phases (Fig 2A, S1 Fig). This is consistent with the known binding profile of Wor1 [25]. Additionally, in white cells Tup1 is associated with the 2.4kb upstream region where Efg1 is known to bind (Fig 2B), and we also found Efg1 in 2 out of 5 Wor1-HBH purifications (S1 Table) [27]. Our observation that Tup1 occupies the *WOR1* promoter preferentially in opaque phase as well as the inability to detect the Wor1-Tup1 interaction without cross-linking further supports the hypothesis that Tup1 interacts with Wor1 on chromatin.

### Tup1 depletion in liquid culture leads to *WOR1* expression, and results in opaque formation when Tup1 is re-expressed

The identification of Tup1, Ssn6 and Tcc1 in all our cross-linked Wor1 purifications prompted us to examine the functions of Tup1 in opaque formation. Tup1 has previously been linked to phenotypic switching [54, 55]. Loss of Tup1 led to a constitutively filamentous cell morphology, and fuzzy and wrinkled colony morphologies. Loss of Tup1 also led to the expression of downstream opaque gene *OP4*, although at a lower level than opaque cells. Additionally, expression of downstream white gene *WH11* remained high in the *tup1* mutants. Therefore, *tup1* mutants express both white and opaque genes [54, 55]. Furthermore, Tup1 depletion and then re-expression led to near complete conversion from white cells to opaque colonies [54]. Most notably, the *tup1* mutants were mating competent, a hallmark feature of the opaque phase [55].

**Fig 3:**
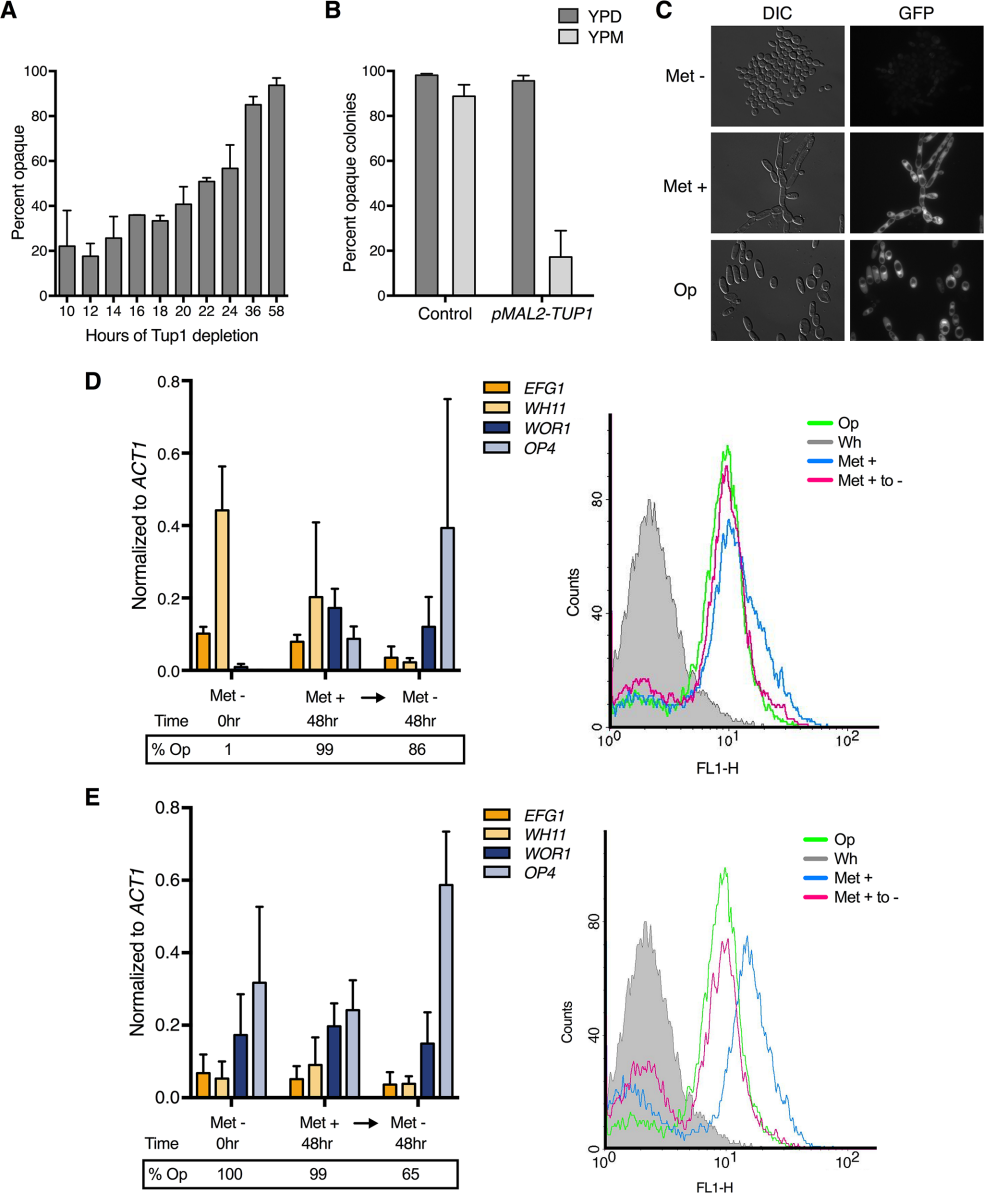
Tup1 is a repressor of the opaque state. **(A)** The effect of Tup1 depletion on opaque formation in liquid medium. White *pMET3-TUP1* (HLY4533) cells were depleted of Tup1 by growth in liquid SCD media containing methionine (Met +) for the indicated times and plated onto SCD media lacking methionine (Met -) plates to assay for opaque cells upon Tup1 re-expression. Plates were grown for 5–7 days at room temperature. **(B)** The effect of *TUP1* overexpression on opaque stability on solid medium. Opaque *pMAL2-TUP1* cells (HLY4537) were grown on SCM plates at room temperature for 5–7 days. Both whole and sectored opaque colonies were counted as opaque. **(C)** *pWOR1-GFP* induction in Tup1 depleted cells. *pMET3-TUP1* cells carrying *WOR1* promoter driven GFP (HLY4535) were cultured in SCD with and without methionine for 48hr at room temperature and imaged for *pWOR1-GFP* expression. Opaque control cells were grown in the absence of methionine. **(D)** Gene expression levels and population dynamics of *pWOR1-GFP* expression in response to Tup1 depletion in starting white cells. White *pMET3-TUP1* cells carrying a *pWOR1-GFP* reporter (HLY4535) were grown in liquid Met + SCD medium for 48hr then washed and transferred to liquid Met—SCD medium and cultured for a further 48hr. Samples for qPCR analysis, plating, and flow cytometry were all taken at the same times, as indicated. The grey curve is starting white *pMET3-TUP1* cells at time 0hr; blue is 48hr in Met+ SCD; pink is 48hr in Met- SCD (Tup1 re-expression); green is an opaque control. **(E)** Gene expression levels and population dynamics of *pWOR1-GFP* expression in response to Tup1 depletion in starting opaque cells. Tup1 depletion procedure is the same as **(D)** except with starting opaque cells. Expression level in **(D)** and **(E)** was normalized to *ACT1* and the average expression level of three independent qPCR experiments are plotted with error bars representing the s.d.

Since these early studies were carried out before the discovery of Wor1 and other key transcriptional regulators of the white-opaque transition, we re-examined the role of Tup1 in white-opaque switching using a conditional *tup1* mutant, as in Zhao et al. [54]. We replaced *TUP1* with a *MET3* promoter-driven *TUP1* in the haploid strain GZY822 [56]. *TUP1* is expressed when grown in the absence of methionine, but repressed upon addition of methionine (S2A Fig). Using this strain, we depleted Tup1 in white cells in liquid cultures, and then plated cells on methionine-containing plates to assay for opaque colonies under the condition of Tup1 re-expression. As had been previously shown in diploid cells [54], extended depletion in liquid Met+ medium followed by re-expression of Tup1 on Met- plates led to near complete conversion of white cells to the opaque phase (Fig 3A). In support of the repressive function of Tup1 in opaque formation, ectopic overexpression of *TUP1* in opaque cells converted them back to the white phase on plates after 5–7 days of growth (Fig 3B). Therefore, Tup1 is a repressor of opaque formation.

Conversion to the opaque phase is slow, with only around 50% opaque formation by 24hr post-shutdown (Fig 3A). These data indicated that Tup1 protein is depleted slowly in the conditional mutant after promoter shutdown. In support of this, we find that Tup1 protein is still detectable at 24hr after promoter shutdown, suggesting that Tup1 protein is highly stable (S2B Fig). Considering that *TUP1* expression levels are similar in white and opaque phases (S1 Fig), and Tup1 protein is highly stable (S2B Fig), we suggest that stochastic phase switching in *C*. *albicans* is not regulated through mechanisms that change Tup1 protein level.

To determine the effect of Tup1 depletion on the expression of Wor1, we introduced a *WOR1* promoter driven GFP reporter construct into the *pMET3-TUP1* conditional mutant strain such that GFP expression correlated with *WOR1* promoter induction. Fluorescent microscopy of white cells cultured in Met + medium at room temperature for 48hr showed cells exhibited robust *pWOR1-GFP* expression on par with opaque control cells (Fig 3C). Therefore, Tup1 depletion induces *WOR1* expression. Since loss of Tup1 leads to a constitutively filamentous phenotype, it is possible that formation of long chains of cells could confound the ability to determine single-cell switching events when cells are plated on solid media. However, we observed uniform GFP induction along Tup1-depleted cell chains and filaments, indicating that all cells in the Tup1-depleted filaments induced similar levels of *WOR1* expression. Therefore, even if an opaque colony did not result from a single cell but instead a filament cluster, all cells in the opaque colony-forming filament cluster would be opaque (Fig 3A).

We next used the conditional mutant reporter strain to assess switching dynamics at the single cell level in liquid culture. White *pMET3-TUP1* cells carrying the *pWOR1-GFP* reporter were grown in liquid Met+ SCD medium for 48hr then washed and transferred to liquid Met- SCD medium and cultured for a further 48hr. Cells were collected for qPCR analysis, plating, and flow cytometry (Fig 3D). Based on the plating data, 99% of white cells had converted to opaque after 48hr of Tup1 depletion and showed expression of the opaque-specific genes *WOR1* and *OP4* (Fig 3D, left panel). While *WOR1* and *OP4* were expressed in the absence of Tup1, the white-phase marker *WH11* was only partially repressed. However, re-expression of Tup1 completely repressed *WH11* expression while increasing *OP4* expression (Fig 3D, left panel). This data is consistent with published findings, with *tup1* cells showing *OP4* induction, although lower than in opaque cells, as well as simultaneous *WH11* expression that is only suppressed upon re-expression of Tup1 [54, 55]. The partial repression of *WH11* and induction of *OP4* in Tup1 depleted cells could be due to an averaging of cell populations in different cell phases. To determine if this was the case, we performed flow cytometry analysis and found that loss of Tup1 led to a single peak of *WOR1* transcription, indicating a single-population response to Tup1 depletion (Fig 3D, right panel), consistent with our earlier fluorescent microscopy findings (Fig 3C). These data demonstrate that Tup1 depletion causes white cells to lose repression of *WOR1* expression, but full expression of *OP4* and repression of *WH11* requires re-expression of Tup1.

We also depleted Tup1 from opaque cells using the same strain and experimental design. Opaque cells depleted of Tup1 remain opaque, both by gene expression in the absence of Tup1 in liquid media and by re-plating assay on methionine-lacking plates (Fig 3E). This unidirectional phase regulation is consistent with a previous report [54]. Therefore, Tup1 depletion in opaque cells does not show further impact on opaque-regulated gene expression. All together, we show that Tup1 depletion in liquid medium can induce *WOR1* expression, leading to an *en masse* white-to-opaque conversion upon Tup1 re-expression. Therefore, Tup1 is a key repressor of the opaque state.

### Tup1 depletion bypasses the requirement for Wor1 in *WOR1* expression

**Fig 4:**
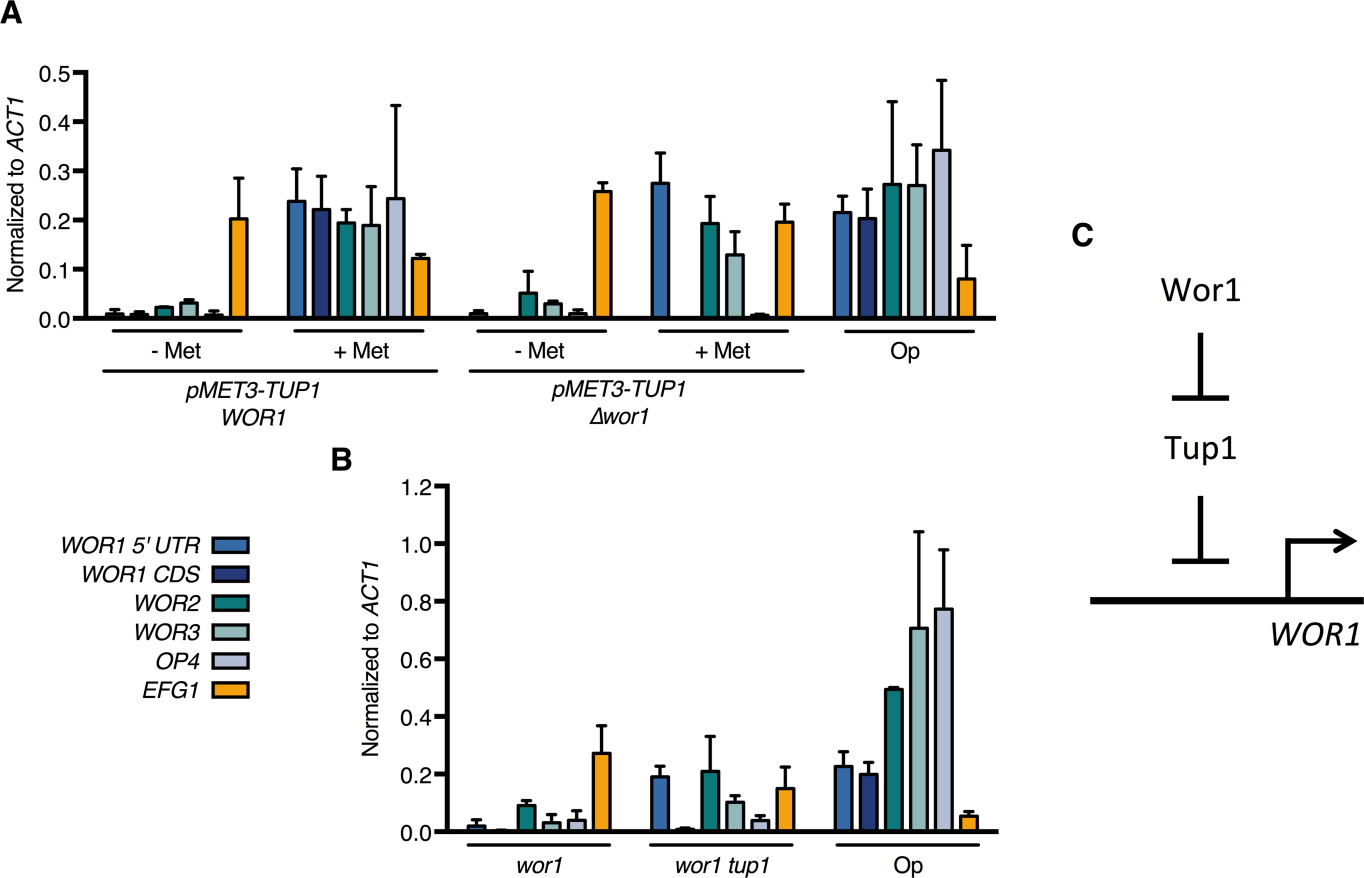
Tup1 depletion bypasses the requirement for Wor1 in the expression of *WOR1* and key opaque regulators. **(A)** Expression levels in the conditional Tup1 mutant and *wor1 pMET3-tup1* conditional double mutant haploid strains. *pMET3-TUP1* (HLY4533) and *pMET3-TUP1 wor1* (HLY4539) were grown at room temperature for 48hr in the presence or absence of methionine. **(B)** Expression levels in diploid *wor1* and *wor1 tup1* mutant strains. Overnight cultures of *MTL****a****/****a*** *wor1* (HLY3570), *wor1 tup1* (HLY4540), and a control strain (JYC1) were inoculated into fresh SCD and grown to log phase. Expression levels of the indicated genes in **(A)** and **(B)** were measured by qPCR and normalized to *ACT1*. *WOR1* expression was measured by qPCR using primers specific to either the *WOR1* 5’ UTR or the *WOR1* coding region (CDS). Average expression level of three independent qPCR experiments was plotted with error bars representing the s.d.**(C)** Genetic model of Wor1-Tup1 regulation of *WOR1* expression.

Since we identified Tup1 as the key repressor of the opaque phase and as Wor1 is the master activator of the opaque phase, we were interested in functional relationship between the two regulators in opaque formation. To address if Tup1 depletion is sufficient to overcome the Wor1 requirement in opaque induction, we deleted the *WOR1* coding sequence in the conditional *pMET3-TUP1* strain. This strain is a conditional *tup1 wor1* double mutant when grown in methionine-containing medium. In addition, the *WOR1* 5’ UTR remained intact in this strain, allowing us to assess transcription from the *WOR1* promoter using primers specific to the 5’ UTR sequence. Depleting Tup1 in the parental *pMET3-TUP1* strain by growth in methionine-containing medium results in the expression of both opaque-specific regulators *WOR1*, *WOR2*, *WOR3*, as well as the opaque specific marker *OP4* (Fig 4A). In the conditional double mutant *pMET3-TUP1 wor1*, Tup1 depletion still induces the expression of the *WOR1* 5’ UTR and other phase regulators *WOR2* and *WOR3*, like the parental *pMET3-TUP1* strain (Fig 4A). Transcription from the *WOR1* coding region is not detected in the conditional double mutant as expected because the CDS region is deleted from this strain. Thus, Tup1 depletion is sufficient to bypass the Wor1 requirement for the expression of *WOR1* and opaque phase regulators.

In order to further validate the results we observed in the haploid conditional double mutant strain, we constructed a *wor1 tup1* double mutant in a diploid background. Using the CRISPR-Cas9 system [57], we deleted *tup1* from a *MTL***a**/**a** *wor1* mutant [22] and examined the expression of opaque genes in the resulting *wor1 tup1* double mutant strain (Fig 4B). Most notably, as in the haploid conditional double mutant, we see induction of *WOR1* 5’UTR expression in the *wor1 tup1* double mutant, but not in the *wor1* single mutant. Additional opaque-specific regulators *WOR2* and *WOR3* are also upregulated in the *wor1 tup1* mutant, although to varying degrees. However, loss of Tup1 in both the haploid and diploid double mutant strains does not induce *OP4* expression, unlike in the *pMET3-TUP1* single deletion strain. This implies that Tup1 does not directly regulate the expression of downstream opaque phase markers, which instead are under the control of Wor1.

This is the first report of a bypass of the master regulator Wor1 in the expression of opaque-specific regulators. This strongly suggests that a major function of Wor1 in opaque formation is to overcome the repressive activity of Tup1 and that Tup1 functions downstream of Wor1 (Fig 4C). This functional relationship predicts that some of the signals known to affect white-opaque switching that are believed to act on Wor1 may work through regulating Tup1.

### Wor1 dissociates from the *WOR1* promoter upon temperature shift to 37°C

**Fig 5:**
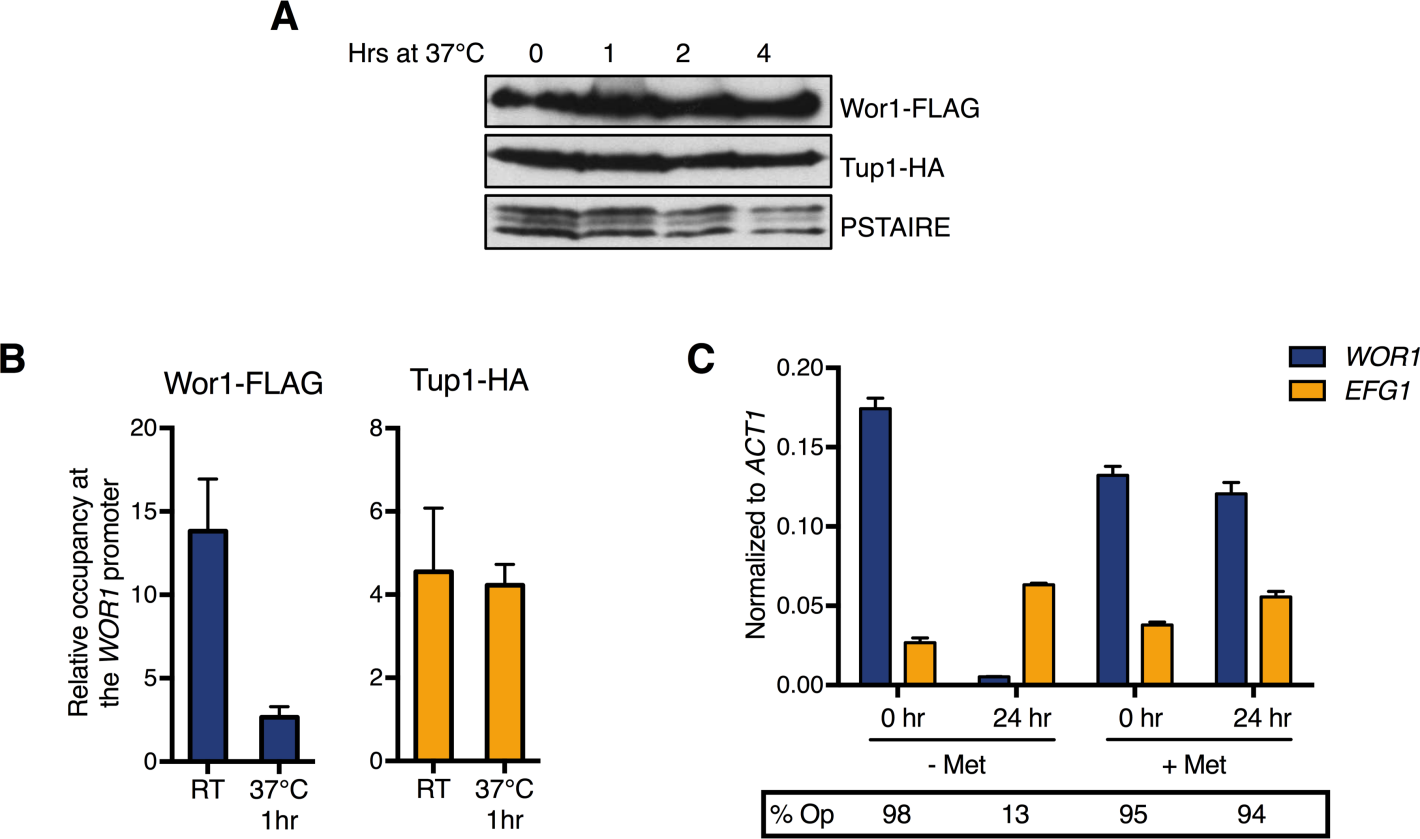
Tup1 depletion stabilizes the opaque state even at 37°C. (**A)** Time course of Wor1 and Tup1 protein levels in opaque cells after shift from room temperature to 37°C. Overnight cultures of opaque cells of a strain carrying both Wor1-FLAG and Tup1-HA (HLY4541) were inoculated, grown to mid-log phase, then shifted to 37°C and grown for the indicated times. Protein level was assessed by Western blot as described. **(B)** ChIP of Wor1-FLAG and Tup1-HA at the *WOR1* promoter in opaque cells shifted to room temperature or 37°C. Overnight cultures of opaque cells of a strain carrying both Wor1-FLAG and Tup1-HA (HLY4541) and an untagged control strain (JYC1) were diluted in SCD and grown to log phase at room temperature. Cultures were divided and incubated at either room temperature or 37°C for one hour, formaldehyde cross-linked, and harvested for ChIP. Enrichment is presented as a ratio of the -4kb region of the *WOR1* promoter IP (bound/input) over an *ADE2* control region IP (bound/input) of the tagged strain, further normalized to the control strain. Values are the average of three independent ChIP experiments with error bars representing the s.d. **(C)** Tup1 depletion in opaque cells at room temperature and 37°C. Opaque *pMET3-TUP1* cells were grown in SCD with or without methionine at either room temperature or 37°C for 24hr. Expression levels of the indicated genes were measured by qPCR and normalized to *ACT1*. Average expression level of three independent qPCR experiments are plotted with error bars representing the s.d. Samples were also taken at the indicated times, washed three times with H_2_O, and plated onto SCD Met- plates to assess phase switching.

One phenomenon regarding phase switching is the complete and synchronous conversion of opaque cells back to the white phase when cultured at high temperatures [58]. Once cells are shifted to 37°C, *WOR1* transcription begins to decrease around 1.5hr, but commitment to the white phase doesn’t occur until around 4–5 hours after temperature shift [59], yet Wor1 protein level is still high at this point (Fig 5A). How *WOR1* transcription decreases when Wor1 protein is still present is unknown. ChIP of Tup1-HA and Wor1-FLAG in opaque cells at room temperature and at 37°C showed that Wor1 binding at the *WOR1* promoter is significantly decreased after just 1hr of temperature shift whereas Tup1 levels remain constant at the *WOR1* promoter (Fig 5B). This dramatic drop in relative levels of Wor1 to Tup1 at the *WOR1* promoter when shifting to 37°C may be the cause of the *en masse* opaque-to-white conversion. To address this, we depleted Tup1 from opaque cells at 37°C for 24hr and assessed opaque phase stability. While wild-type opaque cells converted completely to the white phase after shifting to 37°C, cells lacking Tup1 continued to express *WOR1* at 37°C and remained opaque upon re-plating on methionine-containing media (Fig 5C). These data suggest that loss of Wor1 binding at 37°C leads to Tup1 repression of *WOR1* expression and opaque-to-white switching, highlighting the centrality of Tup1 and providing a mechanism for temperature-induced phase switching.

### Carbon source affects opaque stability and Tup1 level at the *WOR1* promoter

**Fig 6:**
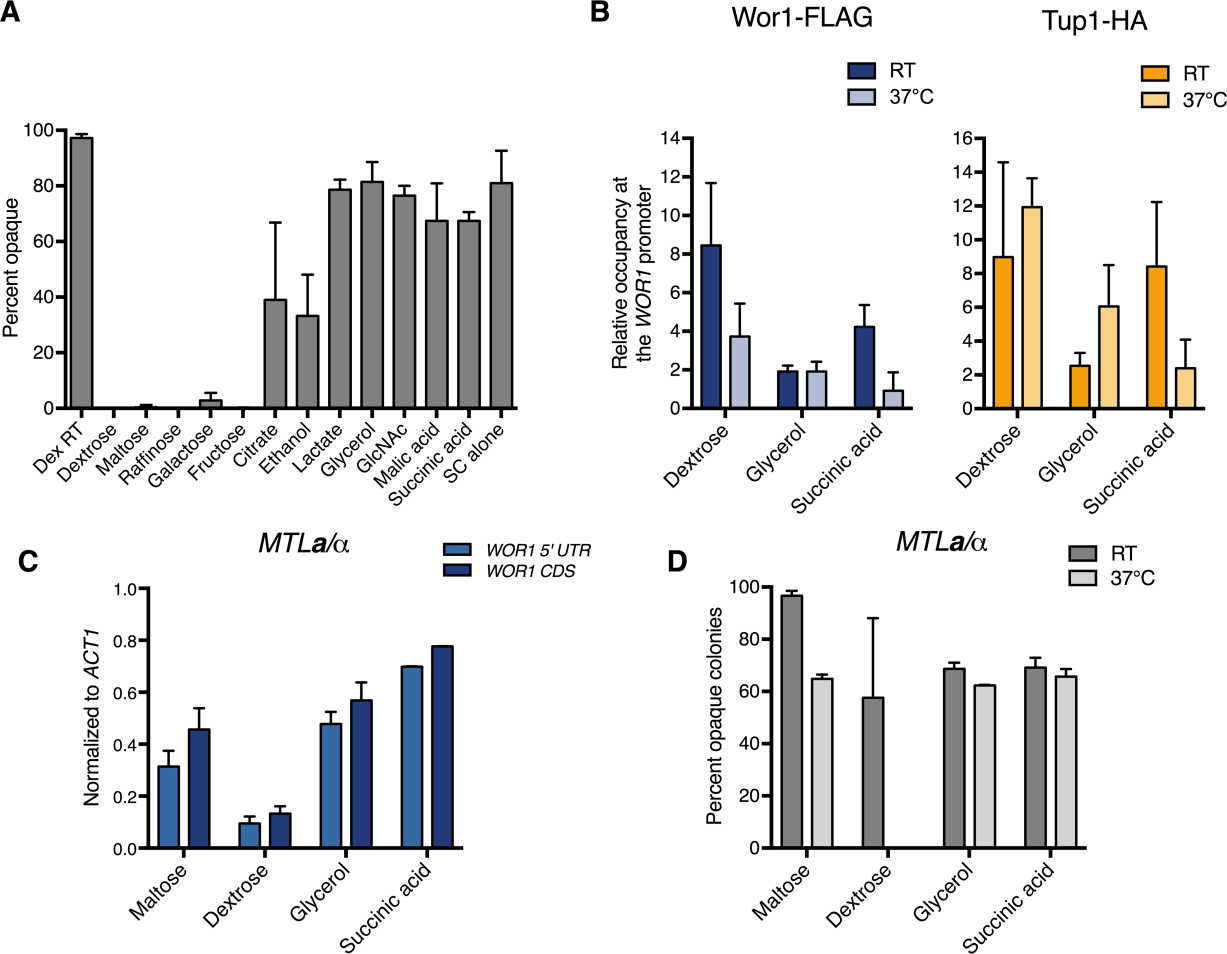
Non-glycolytic carbon sources alter Tup1 occupancy at the *WOR1* promoter and stabilize the opaque phase at 37°C in *MTL*a/a and a/α cells. **(A)** Opaque stability of *MTL***a**/**a** cells cultured in various carbon sources at 37°C for 24hr. Overnight cultures of *MTL***a**/**a** WT opaque cells (HLY3555) grown in SCD were washed three times with H_2_O and inoculated into fresh SC medium containing the indicated carbon sources. Cultures were grown at room temperature for 3hr then transferred to 37°C for 24hr. Samples were plated onto SCD plates and grown for 5–7 days to assess phase switching. **(B)** ChIP of Wor1 and Tup1 in opaque cells at room temperature and 37°C in different carbon sources. Opaque cells carrying both Wor1-FLAG and Tup1-HA (HLY4541) and an untagged strain (JYC1) were grown in SC medium containing the indicated carbon source overnight at room temperature. Cultures were diluted and grown to log phase, then grown at either room temperature or 37°C for 1hr for ChIP. Enrichment is presented as a ratio of the -4kb region of the *WOR1* promoter IP (bound/input) over an *ADE2* control region IP (bound/input) of the tagged strain, further normalized to the control strain. Values are the average of three independent ChIP experiments with error bars representing the s.d. (**C)** Opaque stability of *MTL****a****/α* cells cultured in liquid media at 37°C for 24hr. Overnight cultures of opaque *MTL****a****/α* cells carrying *pMAL2-WOR1* (HLY4543) from SCM were washed three times with H_2_O and inoculated into YNB medium containing the indicated carbon sources. Cultures were grown for 3hr at room temperature then shifted to 37°C. Cells were collected after 24hr and gene expression levels were analyzed by qPCR and normalized to *ACT1*. Average expression level of three independent qPCR experiments are plotted with error bars representing the s.d. **(D)** Opaque stability of *MTL****a****/α* cells on solid media. Overnight cultures of opaque *MTL****a****/α* cells carrying *pMAL2-WOR1* (HLY4543) grown in SCM were washed three times with H_2_O then plated onto YNB plates containing 2% of the indicated carbon source. Plates were incubated at room temperature or 37°C for 5–7 days and scored for percent opaque. Both whole and sectored opaque colonies were counted as opaque.

While high temperature drives conversion to the white state, opaque cells occur naturally within the human host, which has an ambient temperature of 37°C. How opaque cells are able to exist at this non-permissible temperature is not explained in the current model. Due to the niche specific optimization of *C*. *albicans*, we postulated that some external signal encountered in the host might stabilize the opaque state at 37°C. Opaque-like GUT cells are induced through passage in the mammalian GI tract, where glucose is low and alternative carbon sources such as GlcNAc and other derivatives of host and bacterial metabolism are present [20, 60]. In opaque cells, genes involved in glycolysis and glucose utilization are downregulated, while genes important in respiration, gluconeogenesis, and fatty acid metabolism are upregulated [14]. Since opaque cells are metabolically hardwired to thrive in non-glucose conditions, we wondered if growth on alternative carbon sources might stabilize the opaque phase at high temperatures. We compared the effect of 12 different carbon sources in SC medium or SC alone, with amino acids as the sole carbon source, on opaque stability at 37°C for 24hr. We found that opaque cells cultured in the presence of the non-glycolytic carbon sources citrate, ethanol, lactate, glycerol, malic acid, succinic acid, and amino acids were able to maintain the opaque state. In contrast, opaque cells were not stabilized in the presence of glucose, maltose, raffinose, galactose, or fructose, which are all metabolized by glycolysis (Fig 6A). Since the stability assay was performed in SC media containing amino acids, which are non-glycolytic, the opaque state is destabilized by the presence of glycolytic carbon sources. As previously observed, GlcNAc was also able to stabilize opaque cells at 37°C despite being a glycolytic carbon source [3]. However, GlcNAc is a potent inducer of the opaque phase while the non-glycolytic carbon sources tested here do not induce opaque formation, so it is likely these carbon sources stabilize the opaque phase in a manner distinct from GlcNAc.

Tup1 has been shown to mediate glucose repression, and we find that both Tup1 depletion (Fig 5B) and growth in non-glycolytic carbon sources (Fig 6A) stabilize opaque cells at 37°C [41, 61]. Therefore, we hypothesized Tup1 binding at the *WOR1* promoter is probably reduced in cells cultured in non-glycolytic carbon sources. ChIP of Tup1 and Wor1 in opaque cells grown in glycerol and succinic acid at room temperature and 37°C show that Tup1 binding at the -4kb region of the *WOR1* promoter is decreased compared to dextrose (Fig 6B). Interestingly, Wor1 binding was also decreased in these conditions, both at room temperature and 37°C (Fig 6B). The observed decrease in promoter occupancy was not due to global loss of Wor1 and Tup1, as Wor1 and Tup1 protein levels did not decrease when cultured in the presence of non-glycolytic carbon sources (S3 Fig). Since opaque cells are stable in these culturing conditions, these data suggest that Tup1 level at the *WOR1* promoter is a driving force controlling opaque stability at 37°C. This reflects the importance of the relative levels and activities between the master activator Wor1 and the key repressor Tup1 at the *WOR1* promoter in opaque cell fate determination.

### Non-glycolytic carbon sources stabilize the opaque phase in *MTL*a/α cells at 37°C

Although most clinical isolates of *C*. *albicans* are *MTL***a**/α, GUT cells are unstable upon exit from the mammalian host due to **a**1-α2 repression of *WOR1* transcription [12, 13, 20, 22–24]. The *in vitro* conditions that stabilize *MTL***a**/α opaque cells outside of the mammalian gut are unknown. Since growth in alternative carbon sources was sufficient to stabilize opaque cells at 37°C in *MTL***a**/**a** cells, we tested if it was also sufficient to stabilize the opaque phase in the presence of **a**1-α2 repression. We transformed an **a**/α wild-type strain with *pMAL2-WOR1* to induce the opaque phase through ectopic expression of *WOR1* when cultured in maltose-containing YNB medium, which does not contain amino acids. Opaque **a**/α cells were washed and inoculated into YNB media containing different carbon sources, grown for 3hr to adapt to the carbon source, then shifted to 37°C for 24hr for expression analysis (Fig 6C). The *pMAL2*-*WOR1* construct only contains the *WOR1* coding sequence, allowing measurement of endogenous *WOR1* expression using primers specific to the *WOR1* 5’ UTR. Due to ectopic *WOR1* expression from the *MAL2* promoter, cells grown in maltose were able to maintain endogenous *WOR1* expression, as indicated by qPCR of the 5’ UTR. The higher level of *WOR1* CDS relative to 5’ UTR likely reflects ectopic *WOR1* expression from the *MAL2* promoter. Conversely, cells grown in dextrose, known to repress the *MAL2* promoter, showed greatly reduced endogenous *WOR1* expression, evident by low transcript levels of *WOR1* 5’ UTR and CDS. Importantly, cells cultured in glycerol and succinic acid were able to maintain high endogenous *WOR1* expression. The similar levels of 5’ UTR and *WOR1* CDS indicate the absence of ectopic *WOR1* expression from the *MAL2* promoter. *WOR1* expression implied opaque stability, and this was confirmed on solid media. Using the *pMAL2-WOR1 MTL***a**/α strain, cells induced to the opaque phase through ectopic *WOR1* expression were spread onto YNB plates containing different carbon sources and grown at 37°C (Fig 6D). Opaque cells on maltose plates remained opaque after 5–7 days of growth at 37°C due to maltose-induced ectopic *WOR1* overexpression. Opaque cells plated on glycerol and succinic acid were also able to maintain the opaque phase at 37°C, while cells plated on dextrose converted completely to the white phase (Fig 6D). Therefore, non-glycolytic carbon sources promote opaque stability at 37°C even in the presence of **a**1-α2 repression. This effect does not extend to all **a**1-α2 repressed genes, as opaque **a**/α cells are unable to mate [22].

## Discussion

**Fig 7:**
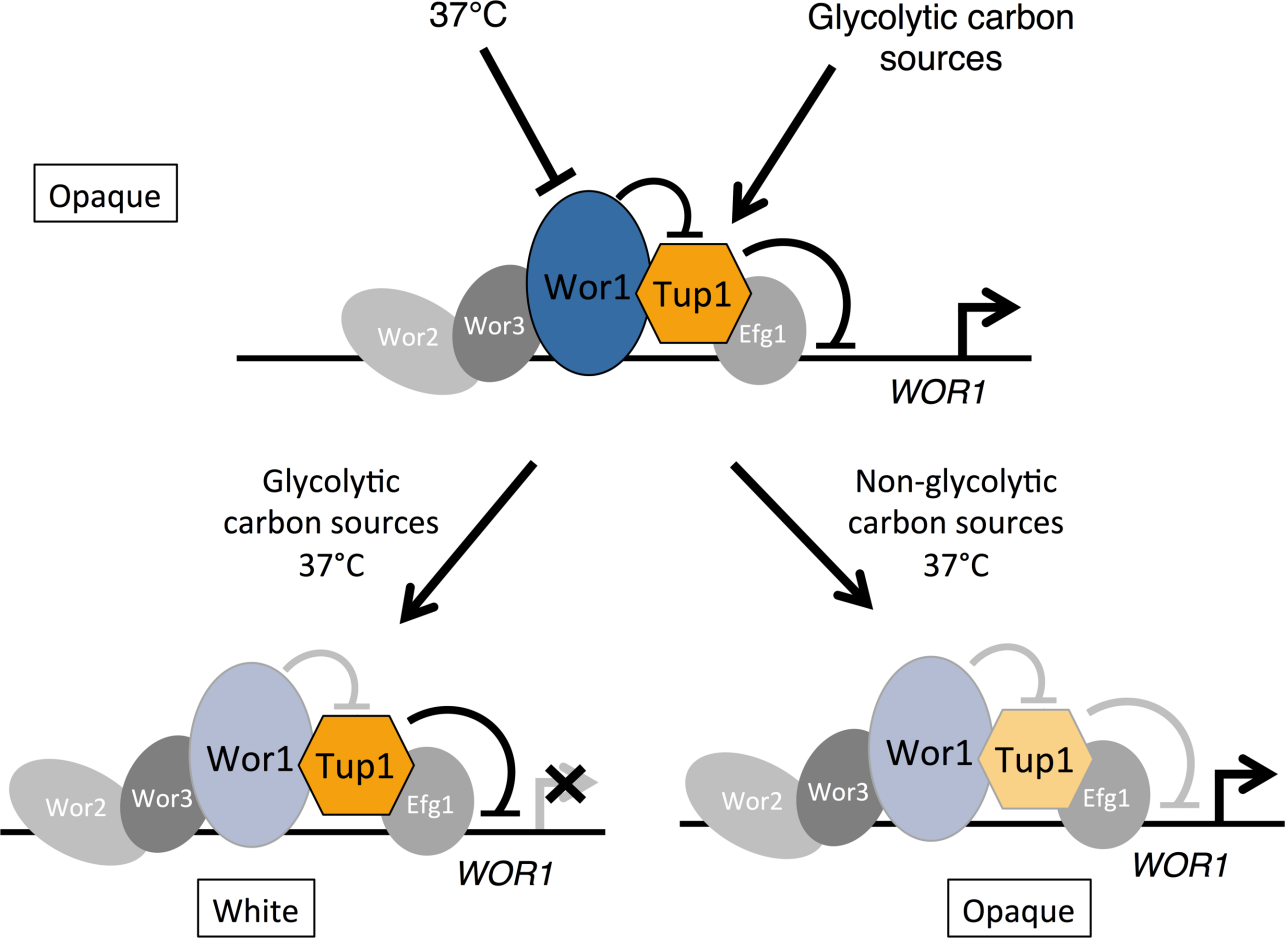
A model for Tup1-mediated regulation of cell fate at the *WOR1* promoter. Tup1 associates at the *WOR1* promoter and represses *WOR1* expression. In opaque cells, Wor1 binds along the *WOR1* promoter and inhibits Tup1-mediated repression of *WOR1* expression. The effect of temperature and carbon source on Wor1 and Tup1 occupancy at the *WOR1* promoter is shown. Dark blue or yellow indicates high levels of Wor1 and Tup1, while light colors depict decreased Wor1 or Tup1 at the promoter. Grey circles depict several other well-characterized phase regulators. Grey lines indicate reduced or lost activity.

Here we have identified Tup1, and Tup1 complex proteins as the major proteins associated with the master regulator Wor1. Our genetic analyses demonstrate that Tup1 is a key repressor of the opaque phase (Fig 7). Tup1 depletion and re-expression leads to complete white-to-opaque conversion, and importantly, Tup1 depletion is able to bypass the requirement for Wor1 in opaque phase establishment and maintenance. Therefore, we propose that Wor1 functions as the master regulator of the opaque switch by inhibiting Tup1-mediated repression of *WOR1* expression (Fig 7). We further show that levels of Wor1 or Tup1 at the *WOR1* promoter in opaque cells are regulated by temperature and carbon source, conditions that control opaque stability. We propose that external signals regulate Wor1 and Tup1 levels and activities at the *WOR1* promoter, and that these relative levels at the promoter control *WOR1* expression and determine cell fate (Fig 7). This study defines Wor1 and Tup1 as the central circuit for opaque phase transcription. Our model illustrates how external cues are interpreted to govern white-opaque switching.

The HBH tandem affinity purification system, utilizing first a Ni^2+^ purification followed by a streptavidin purification, is designed to purify proteins in denaturing conditions [44]. This system, combined with formaldehyde crosslinking, allows capturing of proteins with weak or transient interactions. Denaturing conditions used throughout the purification also reduce protein degradation and PTM loss. Indeed, the strength of this method was readily observed when we were unable to recapitulate the Wor1-Tup1 interaction by immunoprecipitation in native conditions absent of any cross-linkers. Ssn6, part of the Tup1 complex, was recently found to overlap with about 40 percent of Wor1-bound promoter regions [62]. The significant overlapping of Wor1 and Ssn6 on promoter regions by ChIP-chip is consistent with Tup1, Ssn6 and Tcc1 being the major proteins found cross-linked to Wor1 in our purifications. However, the incomplete pattern of genome-wide co-occupancy of Wor1 and Ssn6 suggests that Wor1 does not recruit Ssn6/Tup1 to chromatin, as we would expect a more complete overlap of binding sites if that was the case. Instead, the Tup1 complex is likely recruited to different genomic regions by additional factors involved in the opaque circuit, such as Efg1, which co-occupies the same 2.4kb upstream region of the *WOR1* promoter as Tup1 in white cells. Interestingly, we did not identify Wor2 in our Wor1-HBH purifications, even though it has been shown to associate highly genome-wide with Wor1 by ChiP-chip [27]. This could be due to several reasons, including proximity to Wor1 on chromatin. So while HBH purification can be effective for identifying chromatin-mediated protein interactions, it is a method distinct from genome-wide ChIP. We believe it can be used to identify key cell fate regulators that function together in other systems.

A large body of work in the field has focused on the identification of new regulators of white-opaque switching, such as Efg1, Wor1, Wor2, Wor3, Czf1, and others [22–28, 62, 63]. Among these regulators, Wor1 is essential for opaque formation. Previous genetic studies have placed Czf1, Wor2 and Wor3 upstream of Wor1. Ectopic Wor1 expression bypasses *wor2* [25], *wor3* [26] and *czf1* [25] mutants in opaque formation, and overexpression of any of these regulators cannot bypass the *wor1* mutant [25, 26]. In contrast, Efg1 may function downstream of Wor1 as a *wor1 efg1* double mutant can become opaque under certain conditions [64]. Genome-wide ChIP studies of central transcription factors show they share similar opaque-enriched binding patterns along each other’s promoter regions [27]. One confounding finding, however, is that both positive and negative regulators, such as Efg1 which has reduced expression in opaque cells, show enriched promoter binding in opaque cells, notably at the 8kb-long, active *WOR1* promoter. Our biochemical approach identified Tup1, Ssn6, and Tcc1 as the major transcriptional regulators associated with Wor1. Wor3 and Efg1 were also found in our Wor1-HBH purifications, consistent with their overlapping occupancy at key opaque promoter regions with Wor1 (Fig 7). We further demonstrated that Tup1 is a key repressor of the opaque state. Tup1 depletion led to the expression of key opaque phase regulators, such as Wor1 and Wor3, but not of opaque-specific genes like *OP4*. Importantly, Tup1 depletion could bypass the Wor1 requirement for opaque phase formation. Recently, Ssn6 has also been identified as a repressor of the opaque cell type [62]. Unlike *tup1*, *ssn6* could not bypass *wor1* in opaque formation, likely due to a redundancy in function of the *Candida*-specific Ssn6 homolog Tcc1 [50]. As Tup1 does not bind DNA directly, it is likely recruited to promoter regions through interactions with other regulators, such as Efg1 (Fig 7). However, *efg1* cells can exist in the white state [65]. Therefore additional regulators are involved in recruiting Tup1 to repress key opaque circuit transcription. How Wor1 functions to inhibit Tup1-mediated repression of transcription is not clear. A similar phenomenon has been reported in *S*. *cerevisiae* regarding Tup1 repression of osmotic stress response genes, where Tup1 remains associated at active promoters upon stress activation [66]. Like Tup1 complexes, Taf14 was identified in all Wor1-HBH purifications and may play a role in transcription initiation and chromatin modification of the phase regulator genes [52, 67]. Our work presents an explanation by means of a previously unidentified function of Wor1 in inhibiting Tup1-mediated repression. The central Wor1-Tup1 regulation allows for the integration of previously identified transcription factors in the white-opaque circuit, both activators and repressors, in controlling *WOR1* expression, with different regulators responding to different environmental cues. Multiple such regulations on the *WOR1* promoter ensure opaque stability, and transient alteration in expression of *WOR1* or other regulators is insufficient to convert opaque cells to the white phase.

A recent study has investigated the effects of a multitude of conditions on morphological programs [68]. Our study investigated two growth conditions, temperature and carbon source. Growth at 37°C converts opaque cells to white *en masse* in glucose-containing medium; correspondingly, Wor1 dissociates from the *WOR1* promoter while Tup1 level remains constant. Tup1 depletion and growth in non-glycolytic carbon sources both stabilize the opaque phase even at 37°C; correspondingly, Tup1 level at the *WOR1* promoter decreases in non-glycolytic carbon sources. We suggest that these two conditions are being integrated at the promoter chromatin to regulate the ratio of Wor1 and Tup1 level (and activity) at the *WOR1* promoter to control *WOR1* transcription and commitment to the opaque state. Temperature-dependent Wor1 binding could be regulated by Hsf1 and Hsp90 orchestrated chromatin remodelling [69]. Tup1 has previously been linked to regulation of carbon metabolism[41], and this and other glucose sensing pathways may control Tup1 occupancy or activity at the *WOR1* promoter. Multiple pathways are expected considering the observed differences in promoter-associated Tup1 and Wor1 between glycerol and succinic acid. The mechanistic details of these regulations require future experiments. One important finding is that lack of glycolytic carbon sources can maintain opaque stability at 37°C even in *MTL***a**/α cells where *WOR1* expression is repressed by **a**1-α2. The capacity of alternative, non-glycolytic carbon sources to stabilize opaque *MTL***a**/α cells at the physiologically relevant temperature of 37°C may explain the ability of opaque or GUT cells to exist in the host gastrointestinal tract, where glucose is low and non-glycolytic carbon sources are present in abundance. Additionally, growth on non-glycolytic carbon sources confers increased resistance to antifungals and other stresses [70] as well as increased virulence both *in vitro* and *in vivo* [70–72]. Therefore, the link between metabolism and cell state is of keen interest, and our work provides one mechanism by which carbon source and cellular metabolism can be sensed and integrated to contribute to the regulation of cell fate.

Both Wor1 and Tup1 are highly conserved fungal regulators. Tup1 regulates hundreds of genes in *C*. *albicans* [41, 73] and has also been implicated in regulating metabolism, stress response, dimorphism, and virulence in a diverse range of pathogenic fungi, including the plant pathogens *Ustilago maydis* and *Magnaporthe oryzae* as well as the clinically relevant human pathogens *Aspergillus fumigatus* and *Cryptococcus neoformans* [74–77]. Wor1 homologs in other fungi have been found to regulate developmental programs such as filamentation, sporulation, and virulence [33, 34, 78]. In *C*. *albicans*, Tup1 is a repressor of both hyphal development and the opaque state. Here we show that Wor1 functions as the master regulator of opaque cell fate by inhibiting Tup1 repression to induce *WOR1* expression and the opaque phase. The same Wor1-Tup1 regulatory circuit may be used widely in other fungi. In *C*. *neoformans* Wor1-homolog Liv3 and CnTup1 are both shown to regulate a gene required for quorum sensing and virulence [77, 79]. In *U*. *maydis*, UmTup1 represses the expression of the Wor1-homolog Pac2, and both proteins regulate filamentation and virulence. Importantly the *U*. *maydis tup1 pac2* double mutant resembles the *tup1* single mutant in gene expression and pathogenic phenotype [74]. These examples support the idea that the Wor1-Tup1 regulatory circuit is conserved across diverse fungi.

## Materials and methods

### Media and growth conditions

Strains were grown in YEP (1% yeast extract, 2% peptone), YNB medium (0.17% Difco yeast nitrogen base w/o ammonium sulfate, 0.5% ammonium sulfate), or synthetic complete (SC) medium (0.17% Difco yeast nitrogen base w/o ammonium sulfate, 0.5% ammonium sulfate, complete supplement amino acid mixture) plus 2% either dextrose or maltose as indicated. Cells grown for the purpose of HBH purification were grown in media supplemented with 4μM biotin. *pMET3-TUP1* cells were routinely cultured in SC media lacking methionine, and *pMET-*mediated repression was induced by addition of 5mM methionine. White and opaque cells were maintained at 30°C and room temperature, respectively.

### Plasmid and *C*. *albicans* strain construction

All strains constructed and used in this study are listed in S2 Table and all primers used in this study are listed in S3 Table.

#### *pMAL2-WOR1-HBH* plasmid

The plasmid pFA6a-HTB-TRP1 was graciously provided by Dr. Kaiser [44]. Flanking MluI and KpnI sites were added to the 5’ and 3’ ends of the construct, respectively, as well as an additional 6xHis tag to the C-terminal end. The construct was codon-optimized for *C*. *albicans* (conversion of serine-coding CTGs to *Candida* leucines) using a two-PCR process with long primers (primers 1–4 listed in S3 Table) carrying point mutations, and an additional KpnI site was removed using the same process. The final product was digested with MluI and KpnI, and inserted into a MluI and KpnI digested *pMAL2-WOR1*-carrying plasmid (p905) generated from the BES119 vector backbone [80], to yield the final construct *pMAL2-WOR1-HBH* (p1251). The plasmid was digested with AscI for integration at the *ADE2* locus and transformed into *MTL****a****/****a*** wild-type strain JYC5, generating HLY4532 [81].

#### *pMAL2-TUP1-HBH* plasmid

*TUP1* was amplified with primers 5 and 6 adding flanking XbaI and MluI restriction sites to the 5’ and 3’ ends, respectively. The sequence was amplified by PCR, double digested with XbaI and MluI. The *pMAL2-WOR1-HBH* backbone was similarly digested to remove the *WOR1* sequence, purified through gel extraction, and *TUP1* was inserted to become *pMAL2-TUP1-HBH* (p1101). The plasmid was digested with either AscI for integration at *ADE2* (HLY4537) or BglII for integration at *TUP1* and transformed into JYC5, generating HLY4542 [81].

#### *pMET3-TUP1-HIS3* strain

The *HIS1* gene (for selection) and *MET3* promoter were amplified and fused together by PCR (primers 7–10), as described [82]. Flanking sequences of around 250bp of the *TUP1* 5’ upstream region and around 30bp of the start of the *TUP1* gene were also added by PCR to the *HIS1-pMET3* construct. The final PCR product (*5’TUP1-HIS1-pMET3-TUP1*) was transformed into the haploid strain GZY822 (graciously provided by Dr. Berman [56]) and verified for integration.

#### *pMET3-TUP1 pWOR1-GFP* strain

The plasmid *pWOR1-GFP* [22], which contains 3.2kb of the *WOR1* promoter, was digested with AscI for integration into the *ADE2* locus and transformed into the above *pMET3-TUP1* strain (HLY4533) to obtain the haploid HLY4535.

#### pMET3-TUP1 wor1::ARG4 strain

*WOR1* was deleted with *ARG4* in the *pMET3-TUP1* conditional mutant strain (HLY4533) using the method described (primers13-16) [82], with *ARG4* integrated into the *WOR1* coding sequence to generate the haploid HLY4539.

#### *tup1 wor1* strain

*TUP1* was deleted in a previously published diploid *MTL****a****/****a*** *wor1* mutant strain (HLY3555) by CRISPR-Cas9 editing as described to generate HLY4540 [22, 57]. Primers 23 and 24 were used to generate the *TUP1* sgRNA plasmid, and primers 25 and 26 were used for the repair template.

#### *pTUP1-TUP1-3xHA* strain

The 3xHA sequence was amplified by PCR with primers 11 and 12 to replace the HBH tag in the *pMAL2-TUP1-HBH* (p1101) plasmid using the MluI and KpnI sites, resulting in the final construct, *pMAL2-TUP1-3xHA* (p1142). The resulting plasmid was linearized with BglII for integration into the *TUP1* locus of the wild-type *MTL****a****/****a*** strain JYC1 to obtain the diploid strain, HLY4538.

#### pTUP1-TUP1-3xHA pMAL2-WOR1-FLAG strain

The *URA3* marker was replaced with *HIS1* in the *pMAL2-TUP1-HBH* vector (p1143) using BamHI and NotI. The plasmid was then digested with NotI and KpnI to remove the *TUP1* coding sequence and replace it with the *WOR1* coding sequence to create the *HIS1*-carrying plasmid, *pMAL2-WOR1-HBH* (p1144). A 3xFLAG tag was then added to the C-terminal region of the *WOR1* coding sequence using PCR with a long primer (primers 21 and 22). The resulting amplicon was digested with XbaI and KpnI and swapped in place of *WOR1-HBH*. The resulting plasmid, *pMAL2-WOR1-FLAG* (p1162), was digested with HpaI and transformed into the *pTUP1-TUP1-3xHA* strain (HLY4541) and integrated into the *WOR1* locus.

#### pMAL2-WOR1 strain

The plasmid *pMAL2-WOR1* (p880) was digested with AscI and integrated into the *ADE2* locus of the diploid *MTL****a****/α* strain CAI4 to obtain HLY4543 [22, 83].

### HBH purification for mass spectrometry

Protein purification was performed as described by Tagwerker et al., with modifications [44]. 200ml cultures of cells expressing either the C-terminally tagged Wor1-HBH or the untransformed background strain were grown in YPM at room temperature, cross-linked for 10 min by the addition of formaldehyde to a final concentration of 1%, centrifuged at 1,892 x g for 5min, washed twice with H_2_O, and lysed with Buffer A (8M urea, 300mM NaCl, 0.5% NP-40, 50mM sodium phosphate pH 8, 50mM Tris pH 8, 20mM imidazole, 1mM PMSF, 1 Roche Complete EDTA-free protease inhibitor tablet) by five pulses of 20 sec intervals using glass beads and the FastPrep system (setting 5.0). Lysates were collected and centrifuged for 10 min at 16,000 x g. In one experiment an optional sonication step to shear DNA and help extract chromatin-associated protein complexes was incorporated at this point using the same protocol described in the ChIP methods section. Protein concentration was measured and the appropriate volume of Ni^2+^-Sepharose (GE Healthcare) was added to the clarified lysates. Samples and beads were incubated for 6h, then washed with 5-10x bead bed volume twice with Buffer A, twice with Buffer B (8M urea, 300mM NaCl, 0.5% NP-40, 50mM sodium phosphate, 50mM Tris, final pH 6.3), and eluted twice with 2.5x bead bed volume of Buffer C (8M urea, 200mM NaCl, 50mM sodium phosphate, 10mM Tris, 2% SDS 10mM EDTA, 300mM imidazole, final pH 4.3). Eluate pH was neutralized by addition of 10% eluate volume of 1M Tris pH 8, and samples were incubated overnight with streptavidin-conjugated agarose beads (Thermo) pre-washed in Buffer D (8M urea, 200mM NaCl, 100mM Tris pH 8, 2% SDS). Samples were then washed with 5-10x bead bed volume twice with Buffer D, twice with Buffer E (8M urea, 200mM NaCl, 100mM Tris pH 8, 0.2% SDS), twice with Buffer F (8M urea, 200mM NaCl, 100mM Tris pH8), and five times with 50mM NH_4_HCO_3_. At all steps, samples were collected and saved for Western blot verification of successful purification. After final washes, samples were trypsin digested overnight at 37°C. Formic acid was added to a final concentration of 1% to stop the digestion and the supernatant was collected. Beads were washed three times with half the bead volume of 25% acetonitrile and 0.1% formic acid, and the supernatants were collected and pooled each time. Samples were concentrated with a SpeedVac and washed twice with 100μl H_2_O before final resuspension in 10μl H_2_O and analysis by mass spectrometry.

### Mass spectrometry and data analysis

LC-MS/MS analysis was carried out for peptide samples using an EASY NanoLC 1000 system (ThermoFisher Scientific) coupled on-line with an LTQ-Orbitrap XL MS (ThermoFisher Scientific) as described [84]. Each cycle of an MS/MS experiment includes one MS scan in FT mode (350–1400 m/z, resolution of 60,000 at m/z 400) followed by data-dependent MS2 scans in the LTQ with normalized collision energy at 35% on the top ten peaks. Raw data were searched against a database consisting of Candida proteins (PA.orf_trans_all_assembly_21_2009_0306_v2 with a total of 6243 protein entries) using MaxQUANT. The mass tolerance for parent ions and fragment ions were set as ± 20 ppm and 0.5 Da, respectively. Trypsin was set as the enzyme, and a maximum of two missed cleavages were allowed. Protein N-terminal acetylation and methionine oxidation were selected as variable modifications. Protein and PSM FDR was set as 0.01. LFQ and iBAQ protein quantitation were calculated using a minimum ratio count of 2 peptides [46, 47]. All raw data was deposited and can be accessed at ftp://MSV000081642@massive.ucsd.edu (username, if prompted: MSV000081642; password: SA10242017UCI).

### Tup1 depletion and switching assays

*pMET-TUP1* haploid cells were cultured overnight in SCD media lacking methionine to maintain *TUP1* expression. Cells were then washed and used to inoculate two fresh 50 ml cultures, one lacking methionine and the other supplemented with 5mM methionine. Cultures were grown at room temperature over the course of several days, and samples were taken at the time points indicated. Samples saved for qPCR analysis were spun down and flash frozen. Samples for switching assays were washed three times with H_2_O before diluting and plating onto SCD met–plates. Plates were grown up at room temperature for 5–7 days and colonies were counted to determine white-opaque percentages.

### Opaque stability assays

Opaque cells were grown overnight at room temperature in SCD. Cells were diluted into fresh SC media with 2% of the indicated carbon source, cultured for 3 hours, then shifted to 37C for 24hr. Cells were then plated onto SCD plates, grown for 5–7 days at room temperature, and scored for percent opaque. Opaque CAI4 carrying *pMAL2-WOR1* were grown overnight in SCM, washed three times with H_2_O, and either plated onto SC plates of the indicated carbon source and incubated at room temperature or 37°C for 5–7 days and scored, or were inoculated into liquid SC media with the indicated carbon source, cultured for 3 hours at room temperature, then shifted to 37°C. After 24hr, cells from liquid cultures were then spread onto SCD plates and grown at room temperature for 5–7 days then scored. Whole and sectored opaque colonies were counted.

### Western blotting

Cells were collected by centrifugation and lysed with glass beads using a FastPrep at a setting of 5.0 with five pulses of 20 sec intervals. Lysates were clarified by centrifugation at 16,000 x g for 10min at 4°C. Samples were prepared with sample buffer and loaded onto 8% SDS-PAGE gels, transferred to nitrocellulose membranes, and probed for proteins of interest. HBH was detected by a mouse monoclonal antibody against the RGS6H sequence (Qiagen), 13xMyc with a rabbit polyclonal (Santa Cruz), PSTAIRE (Cdc28) with a rabbit polyclonal (Santa Cruz), HA with an HRP-conjugated anti-HA antibody (Roche), and FLAG with a mouse monoclonal (Sigma). All non-HRP-conjugated antibodies were detected by either goat anti-mouse or goat anti-rabbit HRP-conjugated secondary antibodies (Bio-Rad).

### Quantitative RT-PCR

RNA samples were prepared using Qiagen’s RNeasy and On-Column DNase Digestion kits. cDNA was synthesized using a Bio-Rad iScript Reverse Transcription Kit and 1μg total RNA. Quantitiative PCR was performed using Bio-Rad SYBR Green mix on a Bio-Rad iCycler. Primers used are listed in S3 Table.

### Chromatin immunoprecipitation

ChIP was performed as described, with slight modifications [85]: overnight cultures were diluted to an OD_600_ of 0.1 and grown to OD_600_ 1.0. Cells grown in different carbon sources were pre-cultured in medium containing the indicated carbon source before dilution. DNA was sheared by sonication for 6 cycles of 20s at 40s intervals using a Bioruptor (Diagenode) and 1% input samples were saved. Experiments examining multiple regulator binding events were prepared from the same chromatin samples and split prior to immunoprecipitation. The HA-tag was immunoprecipitated using a 12CA5 anti-HA mouse monoclonal antibody (Roche), and the FLAG-tag was immunoprecipitated using anti-FLAG M2 mouse monoclonal antibody (F3165, Sigma). DNA from input and IP samples was quantified by qPCR. Enrichment of tagged samples was quantified by comparing the region of interest IP (bound/input) over the control region *ADE2* IP (bound/input) and further normalizing to an untagged strain, with values representing at least three independent experiments and error bars representing the s.d. Primers used are listed in S3 Table.

### Flow cytometry

Flow cytometry was performed using the BD FACSCalibur system. Cells were passed through cell-strainer caps (BD Falcon) and sonicated prior to flow cytometry analysis. 10,000 cells were counted per sample and measured for GFP expression using the FL1-H channel. FACS data was analyzed using BD CellQuest Pro software.

### Microscopy

Haploid cells carrying *WOR1* promoter driven GFP (HLY4535) were grown in SCD medium with or without methionine at room temperature for 48hr. Images were taken using an inverted Zeiss Axio Observer.Z1 microscope (Carl Zeiss MicroImaging) equipped with an X-Cite series 120 mercury lamp. GFP fluorescence and cell morphology were imaged using the GFP and DIC channels, respectively.

## Supporting information

S1 Table Wor1 interacting proteins identified by mass spectroscopy. Proteins listed are identified only in Wor1-HBH purifications performed with cross-linking, but not in non-cross-linked or untagged purifications. Proteins are ranked in descending order first based off number of times identified in Wor1-HBH cross-linked purifications and then by decreasing average normalized iBAQ (intensity-based absolute quantification) value derived via the MaxQuant suite. Proteins marked by § are known components of the Tup1 co-repressor complex. Proteins marked with an asterisk were also observed in Wor1-HBH purifications without cross-linking.
(XLSX)

S2 Table Strains used in this study. (XLSX)

S3 Table Primers used in this study. (XLSX)

S1 Fig Tup1 levels are similar in white and opaque cells. **A.** *TUP1* expression level in white and opaque cells. Overnight cultures of WT white and opaque cells (JYC5) were inoculated and grown to log phase. *TUP1* expression level was measured by qPCR and normalized to *ACT1*. Expression values are the average of three independent qPCR experiments and error bars represent the s.d. **(B)** Tup1 protein level in white and opaque phases. Overnight cultures of a strain carrying *pTUP1-TUP1-HBH* (HLY4542) were inoculated into fresh YPD, grown to log phase. Protein level was assessed by Western blot as described.
(TIFF)

S2 Fig Verification of *pMET3-TUP1* construct and Tup1 protein stability. **A.** Shutdown of *TUP1* expression by methionine addition in the conditional mutant haploid strain carrying *pMET3-TUP1*. Overnight cultures of white *pMET3-TUP1* (HLY4533) cells grown in Met- SCD were diluted into fresh SCD with and without 5mM methionine. Cells were collected after 24hr and *TUP1* expression level was quantified by qPCR and normalized to *ACT1*. Expression values are the average of three independent qPCR experiments and error bars represent the s.d. **B.** Tup1 protein stability. White cells of a strain carrying *pMAL2-Tup1-Myc* (HLY4536) were grown in YPM overnight, washed with H_2_O, and inoculated into YPD to shut down *pMAL2* promoter activity. Samples were taken at the indicated times and Tup1 protein level was assessed by Western blot, as described.
(TIFF)

S3 Fig Protein levels of Wor1 and Tup1 remain constant when cultured in non-glycolytic carbon sources. Opaque cells carrying both Wor1-FLAG and Tup1-HA (HLY4541) were cultured overnight at room temperature in SC medium with the indicated carbon sources. Mid-log cultures were split then grown for an additional hour at either room temperature or 37°C and harvested. Tup1 and Wor1 levels were assessed by Western blot, as described.
(TIFF)
