# Supplementary figures and images for: Wor1 establishes opaque cell fate through inhibition of the general co-repressor Tup1 in Candida albicans
Source: PLoS Genet. 2018 Jan 16;14(1):e1007176. doi: 10.1371/journal.pgen.1007176 (PMC5786334; doi:10.1371/journal.pgen.1007176)

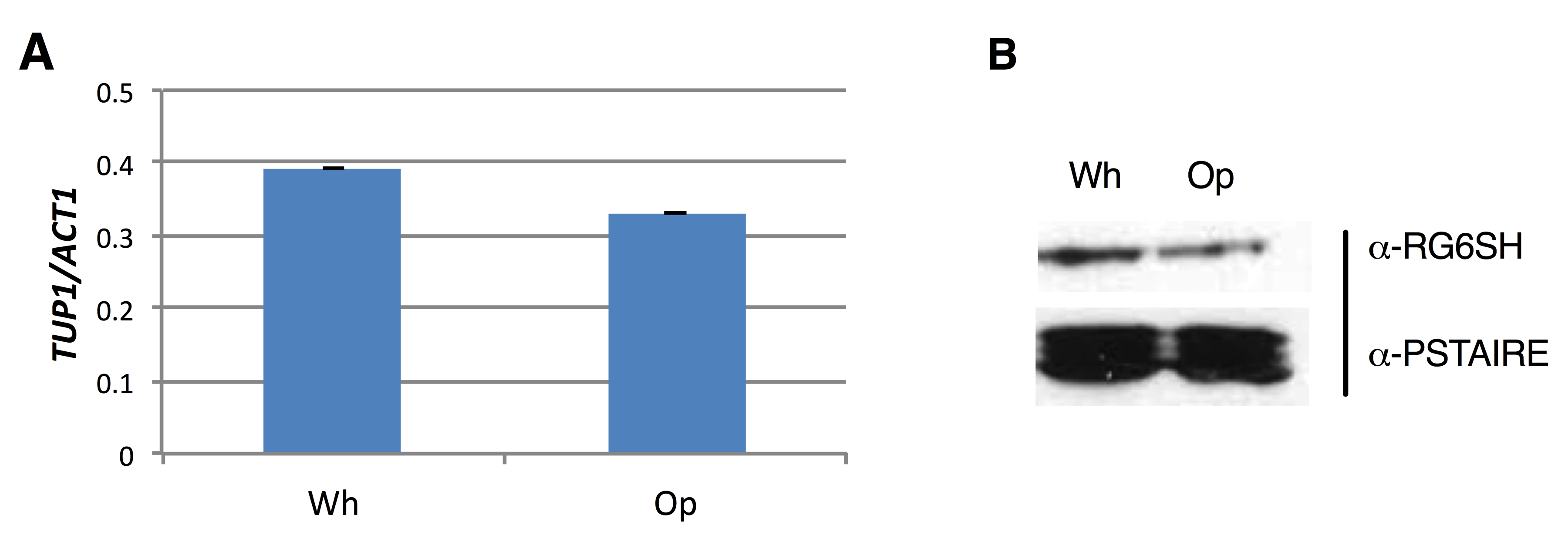

Supplement: S1 Fig — A. TUP1 expression level in white and opaque cells. Overnight cultures of WT white and opaque cells (JYC5) were inoculated and grown to log phase. TUP1 expression level was measured by qPCR and normalized to ACT1. Expression values are the average of three independent qPCR experiments and error bars represent the s.d. (B) Tup1 protein level in white and opaque phases. Overnight cultures of a strain carrying pTUP1-TUP1-HBH (HLY4542) were inoculated into fresh YPD, grown to log phase. Protein level was assessed by Western blot as described. (TIFF) [file pgen.1007176.s004.tiff]

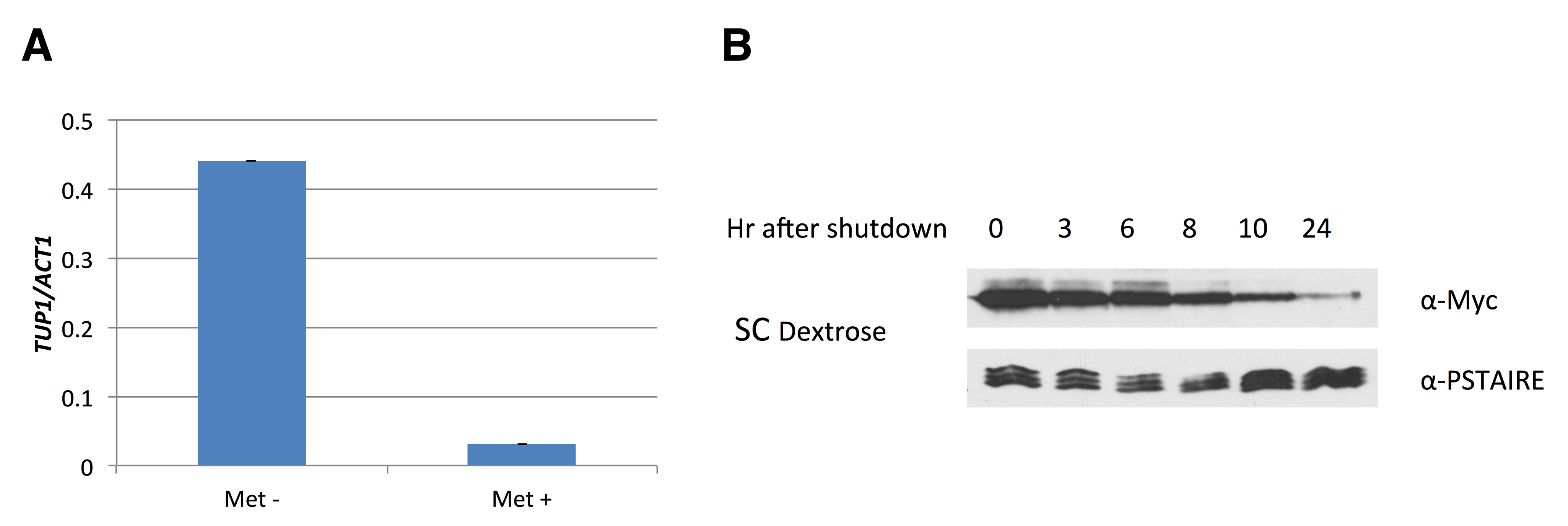

Supplement: S2 Fig — A. Shutdown of TUP1 expression by methionine addition in the conditional mutant haploid strain carrying pMET3-TUP1. Overnight cultures of white pMET3-TUP1 (HLY4533) cells grown in Met- SCD were diluted into fresh SCD with and without 5mM methionine. Cells were collected after 24hr and TUP1 expression level was quantified by qPCR and normalized to ACT1. Expression values are the average of three independent qPCR experiments and error bars represent the s.d. B. Tup1 protein stability. White cells of a strain carrying pMAL2-Tup1-Myc (HLY4536) were grown in YPM overnight, washed with H2O, and inoculated into YPD to shut down pMAL2 promoter activity. Samples were taken at the indicated times and Tup1 protein level was assessed by Western blot, as described. (TIFF) [file pgen.1007176.s005.tiff]

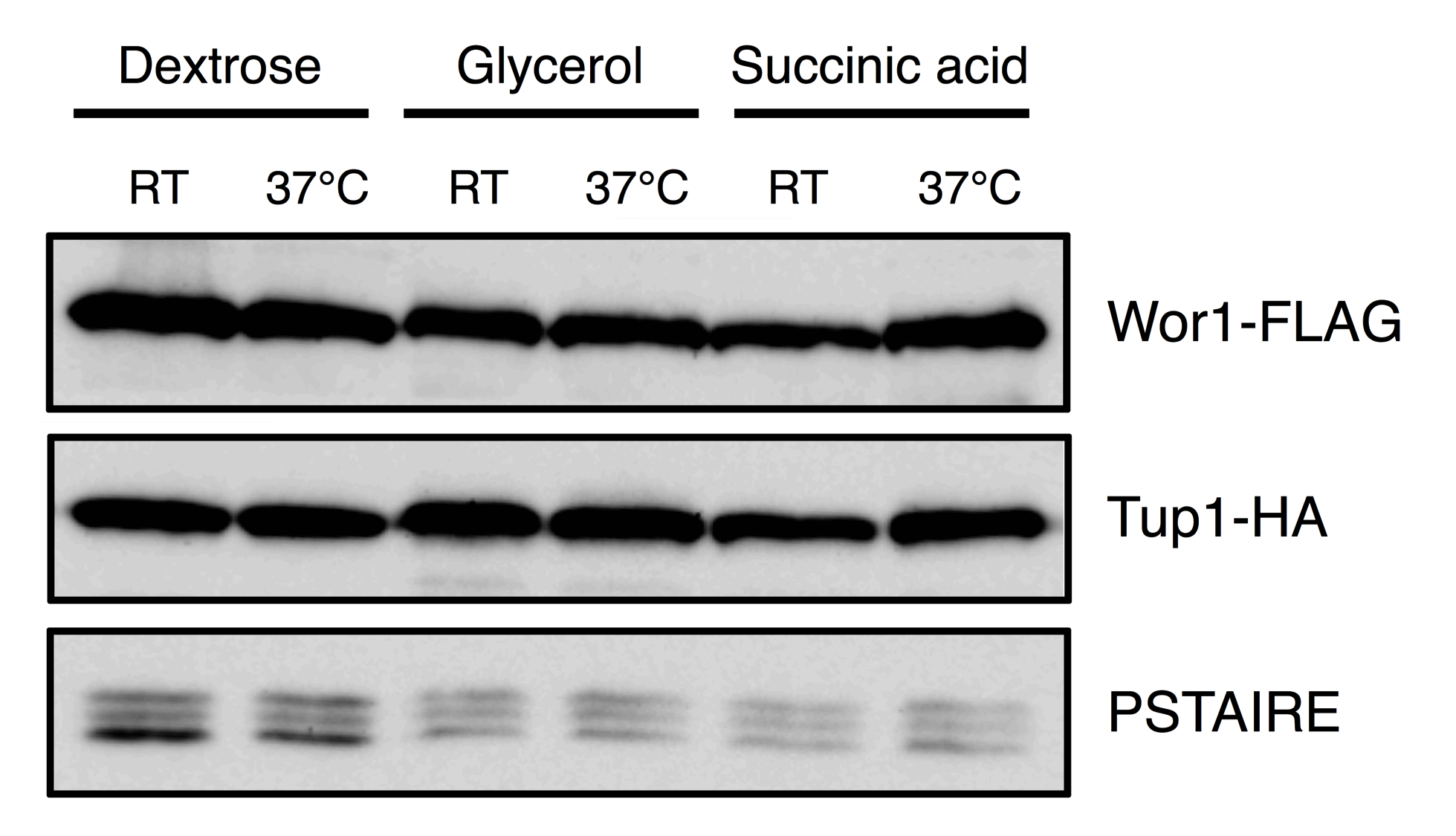

Supplement: S3 Fig — Opaque cells carrying both Wor1-FLAG and Tup1-HA (HLY4541) were cultured overnight at room temperature in SC medium with the indicated carbon sources. Mid-log cultures were split then grown for an additional hour at either room temperature or 37°C and harvested. Tup1 and Wor1 levels were assessed by Western blot, as described. (TIFF) [file pgen.1007176.s006.tiff]
